# Potential Mechanisms of Exercise in Gestational Diabetes

**DOI:** 10.1155/2013/285948

**Published:** 2013-04-09

**Authors:** Saeid Golbidi, Ismail Laher

**Affiliations:** Department of Pharmacology and Therapeutics, Faculty of Medicine, University of British Columbia, Vancouver, BC, Canada V6T 1Z3

## Abstract

Gestational diabetes mellitus (GDM) is defined as glucose intolerance first diagnosed during pregnancy. This condition shares same array of underlying abnormalities as occurs in diabetes outside of pregnancy, for example, genetic and environmental causes. However, the role of a sedentary lifestyle and/or excess energy intake is more prominent in GDM. Physically active women are less likely to develop GDM and other pregnancy-related diseases. Weight gain in pregnancy causes increased release of adipokines from adipose tissue; many adipokines increase oxidative stress and insulin resistance. Increased intramyocellular lipids also increase cellular oxidative stress with subsequent generation of reactive oxygen species. A well-planned program of exercise is an important component of a healthy lifestyle and, in spite of old myths, is also recommended during pregnancy. This paper briefly reviews the role of adipokines in gestational diabetes and attempts to shed some light on the mechanisms by which exercise can be beneficial as an adjuvant therapy in GDM. In this regard, we discuss the mechanisms by which exercise increases insulin sensitivity, changes adipokine profile levels, and boosts antioxidant mechanisms.

## 1. Introduction

Gestational diabetes mellitus (GDM) is the most prevalent metabolic disorder during pregnancy and is defined as glucose intolerance of variable severity that is first diagnosed during pregnancy and usually resolves not long after delivery [1, 2]. This definition includes any degree of glucose intolerance from just impaired to frankly diabetic [3]. Resolution of the condition is also important when differentiating between previously undiagnosed type 2 diabetes and GDM [4]. Insulin resistance, due to a series of hormonal changes, contributes to decreased blood glucose uptake by muscles [5]. This phenomenon seems to be important from an evolutionary point of view, as it ensures adequate glucose supply for fetal growth and development. In the third trimester a healthy pregnant woman has to increase her insulin secretion by 2–4 times to maintain glucose levels within normal limits. Pregnant women who develop GDM are unable to augment insulin production to compensate for their increased resistance to insulin [6].

There are several modifiable and unmodifiable risk factors for developing GDM. Obesity is a modifiable risk factor that is strongly associated with the development of gestational diabetes. In a survey of 97000 singleton births, obese women had a 3-fold increased risk of developing GDM than nonobese women [7]. Not only obese (body mass index (BMI) > 30 (kg/m^2^)) but also overweight women (29 ≥ BMI ≥ 25 (kg/m^2^)) have a 1.8 to 6.5 times greater risk of developing GDM [8]. It is important to appreciate that there are parallel increases in both obesity and GDM, making it difficult to determine the contribution of obesity as an independent risk factor. The Hyperglycemia and Adverse Pregnancy Outcome (HAPO) study reveals a direct relationship between BMI and pregnancy complications (preeclampsia, caesarian section, higher neonatal birth weight) in pregnant women. This study also reported that maternal plasma glucose correlates with adverse pregnancy outcomes [9]. A study by Getahun et al. reports a significant increase in the prevalence of GDM from 1.2% to 4.2% in between 1989 and 2004 [10]. In the United States, GDM affects 14% of all pregnancies, causing approximately 200,000 cases annually [11]; however, its prevalence varies widely (1.7%–11.6%) between racial and ethnic groups [12]. Recently, the International Association of Diabetes and Pregnancy Study Groups recommended new screening criteria for GDM based on the HAPO study. Using these criteria, the total incidence of GDM reaches almost 18% percent [13]. In Canada GDM is diagnosed in 3.7% of nonaboriginal and 8%–18% of first-nations pregnancies [14]. Another meta-analysis study showed that the risk of developing GDM was 2.14-, 3.56-, and 8.56-fold higher in overweight, obese, and severely obese pregnant women [15]. The diagnosis of GDM is associated with increased body fatness as indexed by prepregnancy BMI; each unit increase in BMI raises the prevalence of GDM by 0.92% [16].

Until a few decades ago, physical activity was discouraged in pregnancy due to myths related to exercise-induced injury and/or adverse fetal and maternal outcomes [17]. However, findings from clinical and epidemiological studies show no adverse maternal and fetal effects on women engaged in mild and moderate physical activities. Indeed, pregnant women are now advised to engage in regular aerobic exercise in the absence of medical or obstetric complications [18]. The American College of Obstetricians and Gynecologists and the American Diabetes Association (ADA) recognize exercise as “a helpful adjunctive therapy” for GDM and suggest 30 minutes or more of moderated exercise a day on most, if not all, days of the week [19, 20]. This paper examines some of the most important pathophysiologic aspects of GDM and discusses how aerobic exercise can benefit some of the physiological adaptations of GDM. 

## 2. Pathophysiology of GDM

Normal pregnancies are associated with increased insulin resistance, which begins in mid pregnancy and continues until delivery. This resistance is thought to be compensated by a nearly 200% to 250% increase in insulin secretion during pregnancy [21]. GDM can be considered as a transient form of type 2 diabetes, with the rapid onset triggered by the metabolic and hormonal changes of pregnancy. Indeed, the same set of underlying causes that induce diabetes, including autoimmune interactions with the pancreatic beta cells and monogenic causes of diabetes and insulin resistance of peripheral tissues, are also involved in the pathogenesis of GDM [22]. Some have even considered GDM “diabetes in evolution.” It is likely that chronic insulin resistance has already developed in most (but not all) GDM patients before conception and that additional insulin resistance occurs during pregnancy [23]. In the long term, chronic insulin resistance and hypersecretion are likely to lead to beta cell dysfunction.

Autoimmune mechanisms may be principle underlying pathophysiologic pathway in a minority (≤10%) of GDM patients. Circulating antibodies against pancreatic beta cells or beta cell antigens (such as GAD) have been detected in GDM patients: insulin deficiency due to immunologic beta cell destruction is the initial step in this group of patients who have evolving type 1 diabetes [24]. The role of pregnancy as an inducer or accelerator of immunologic damage is yet to be determined.

A monogenic form of diabetes constitutes 1%-2% of all GDM patients, who either have an autosomal dominant mutation (sometimes referred to as maturity-onset diabetes of the young (MODY)) or a mutation in mitochondrial DNA (which is often associated with deafness). Such patients with preexisting disease are usually diagnosed during pregnancy screening tests. There is no direct correlation between BMI and the monogenic form of GDM, as patients tend not to be obese or have insulin resistance. The main underlying pathophysiology is dysregulation of beta cell mass or function which results in hyperglycemia. Several subtypes of MODY have been described in women with GDM, including MODY2 (mutation in glucokinase gene), MODY3 (mutation in hepatocyte nuclear factor 1*α*), and MODY4 (mutation in insulin promoter factor 1) [22].

**Figure 1:**
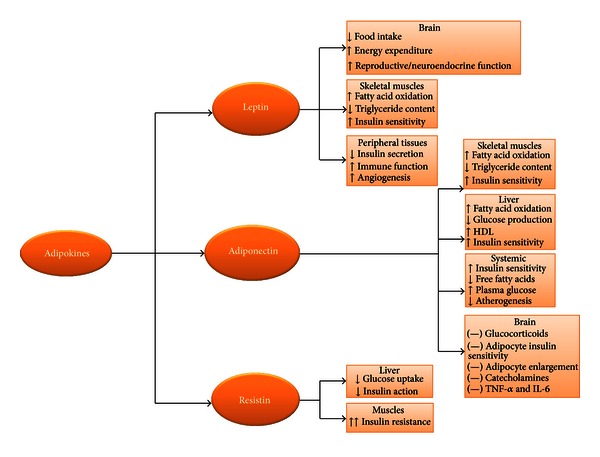
Selected physiologic roles of adipokines in relation to glucose metabolism and insulin sensitivity (↑increase, ↓decrease, (—) inhibit).

The mechanisms of pregnancy-induced insulin resistance are not clear but variations in steroid and/or lactogenic hormone levels may have some role. In particular, human placental lactogen, human placental growth hormone, progesterone, cortisol, and prolactin are known to counteract the effects of insulin [25]. This is supported by some evidence such as (i) the chronology between raised insulin resistance and the growth of fetoplacental unit which is accompanied by increased production of these hormones, (ii) the similarity of metabolic changes after administration of these hormones to nonpregnant individuals having the metabolic dysregulation of GDM, and (iii) impaired glucose uptake after exposure of insulin-sensitive cells such as adipocytes caused by pregnancy hormones [25]. However, changes in hormone concentrations do not directly correlate with insulin resistance and do not imply a simple cause-and-effect relationship [26]. Recent data has focused on the roles of adipose tissue-derived mediators, such as adiponectin, leptin, resistin, tumor necrosis factor-alpha (TNF-*α*), visfatin, apelin, and chemerin in the pathogenesis of insulin resistance and inflammation (Figure 1) [27].

*Adiponectin*

Human adiponectin consists of 244 amino acids and has a distinct domain structure. It has a collagen-like and a globular C1q-like domain (similar to the complement component C1q). This adipokine circulates in the blood in at least three homomeric complexes: trimer (low-molecular weight form, LMW), hexamer (medium molecular weight form, MMW), and higher order multimers (high molecular weight form, HMW) [28, 29]. Plasma concentrations reveal a sexual dimorphism, with females having higher levels than males [30]. The HMW form may be the most biologically active form regulating glucose homeostasis [31, 32], but other studies show that even though the HMW form has a greater association with some cardiovascular diseases [33], it has a similar utility for the identification of insulin resistance and metabolic disturbances as does total adiponectin [34]. As opposed to other adipocytokines, plasma levels of adiponectin inversely correlate with body mass index (BMI), intra-abdominal fat, and indices of insulin resistance [35]. Plasma levels of adiponectin decrease with weight gain and are increased by weight loss [36, 37]. Many studies suggest that adiponectin is an important regulator of insulin sensitivity and glucose homeostasis, with several reports confirming an inverse relationship between insulin resistance and plasma adiponectin levels [38]. Hypoadiponectinemia is also associated with beta cell dysfunction [39, 40]. Other studies show that adiponectin has anti-inflammatory effects, such as inhibition of endothelial nuclear factor kappa B (NF-*κ*B) and suppression of phagocytic activity and TNF-*α* production in macrophages [38, 41, 42]. Adiponectin levels in early pregnancy seem to be unchanged or decreased [43–45] and are inversely related to maternal BMI and insulin sensitivity [46]. However, in GDM pregnancies, adiponectin levels decrease independently of changes in maternal BMI or insulin sensitivity [43, 47–49]. A study by Cseh et al. observed significantly decreased plasma adiponectin levels in 30 women with GDM, compared with 40 nondiabetic pregnant women; they reported that plasma adiponectin levels had a negative linear correlation with serum tumor necrosis factor-*α* (TNF-*α*), leptin, fasting C-peptide concentration, BMI, and fasting C-peptide/blood glucose ratio (which was used as an indirect parameter of insulin resistance) [50]. Furthermore, lower first trimester adiponectin levels were predictive of the development of GDM later in pregnancy. Women with adiponectin concentrations lower than 6.4 *μ*g/mL experience a 4.6-fold increased risk of GDM, compared to those with higher concentrations [51]. A few studies have measured adiponectin levels after delivery at different time intervals (3 months and 1, and 1.5 years) in women with GDM and compared with those women having normal pregnancies. Hypoadiponectinemia persists even 1.57 years after delivery in GDM subjects, where it is associated with decreased insulin sensitivity and low HDL and and negatively correlated to other inflammatory markers such as CRP, plasminogen activator inhibitor-1 (PAI-1), and IL-6, even after adjustment for BMI [52–54]. Even though the basis for hypoadiponectinemia and GDM is unclear, suggested mechanisms for the insulin sensitizing effect of adiponectin include (a) promotion of insulin signaling at the receptor/postreceptor level, (b) reduction of gluconeogenesis, (c) improved lipid oxidation, and (d) inhibition of TNF-*α* signaling in adipose tissue [55]. Some experiments with globular adiponectin, whose *in vivo* importance is questionable, propose a role for AMPK [56] and PPAR*α* [57] in its metabolic effects on skeletal muscles. Muscle binding of adiponectin translocates GLUT4 (resulting in increased glucose uptake) and increases nonoxidative glycolysis while also reducing intramyocellular triacylglycerol content to improve fatty acid oxidation [58, 59]. Adiponectin also sensitizes liver cells to the actions of insulin and suppresses the synthesis and function of enzymes such as phosphoenolpyruvate carboxykinase and glucose-6-phosphatase involved in gluconeogenesis [60]. Adiponectin also affects fatty acid metabolism in the liver with secondary influences on plasma triacylglycerol and circulating nonesterified fatty acids. Adiponectin also induces insulin secretion *in vitro* and *in vivo* [61]. While the information on the influence of adiponectin in normal insulin sensitivity is unclear [62, 63], it does, however, appear to augment insulin secretion during insulin resistance [63]. Several studies report that adiponectin has antiapoptotic effects on beta cells, both in cell culture and islet preparations [64, 65].

*Leptin*

Leptin is a 16kDa protein hormone that plays a key role in regulating energy intake and energy expenditure, including appetite and metabolism. It is one of the best known hormone markers of obesity, and in humans, the leptin gene is located on chromosome 7 [66]. So far, six types of receptors have been recognized for leptin (Ob-Ra-f). Ob-Re does not encode a transmembrane domain and is secreted and circulates in human plasma and represents the major leptin-binding activity [67]. Janus-activated kinase (JAK), signal transducers and activators of transcription (STAT), insulin receptor substrate, and the mitogen-activated protein kinase (MAPK) pathways are important leptin intracellular signaling mechanisms [68]. The binding of leptin to its receptor leads to the formation of the Ob-R/JAK2 complex and activation of STAT3, which is phosphorylated and migrates to the nucleus to presumably effect changes in gene expression [69]. Binding of leptin receptors to JAK2 also results in JAK2 autophosphorylation [70], which in turn phosphorylates insulin receptor substrate proteins, and involvement of phosphatidyl inositol 3-kinase to activate downstream signals [71].

During pregnancy leptin is produced by maternal and fetal adipose tissues, as well as by placental cells [72]. Plasma levels of leptin increase by 150% to 200% in the second and third trimesters over those occurring in the first trimester. Many physiological functions have been attributed to leptin, including regulation of food intake and energy balance through central hypothalamic pathways, signaling to the reproductive system (stimulating secretion of GnRH from hypothalamus, FSH and LH from pituitary gland), inhibition of insulin secretion from pancreatic beta cells, stimulation of glucose transport and utilization, glycogen synthesis, and fatty acid metabolism [73, 74]. Reduction of insulin secretion from pancreatic beta cells can result from the effect of leptin on the ATP-sensitive potassium channels. It has been proposed that leptin prevents beta cell stimulation by blocking cAMP signaling. Furthermore, leptin may hinder insulin secretion through cAMP-dependent protein kinase A (PKA) and protein kinase C (PKC). Leptin also regulates endocrine function, inflammation, immune response, and angiogenesis. Weight loss, fasting, and starvation reduce leptin concentrations, while weight gain and hyperinsulinemia have the opposite effects [75–79]. Plasma levels of leptin in pregnant women are 2- to 3-fold above nonpregnant levels and result from an upregulation of adipocyte synthesis in the presence of insulin resistance and hyperinsulinemia [79]. The origin of pregnancy-induced increases in leptin levels remains unclear [80]. Some evidence implies that the placenta, instead of adipose tissue, is the main site of leptin production; for instance, increased leptin levels precede increases in maternal weight [81]. The human placenta also has high leptin mRNA content [82]. Furthermore, maternal leptin levels drop after delivery. More than 90% of placental leptin is released to the maternal circulation [81]. Leptin also has many functional roles in the human fetus, including embryonic implantation, developmental growth, and organogenesis. For instance leptin plays critical roles in the development of the fetal skeletal and lung development.

Many studies document increased maternal leptin levels in GDM [83–86] and hyperleptinemia in early pregnancy, which may have predictive implications. In a study of 823 pregnant women in early pregnancy (13 weeks), Qiu et al. found a strong linear association between maternal plasma leptin concentration and the risk of GDM later in pregnancy. After adjusting for maternal prepregnancy adiposity and other confounders, those subjects with leptin concentration of 31.0 ng/mL had a 4.7-fold increased risk of GDM compared to those who had concentrations of 14.3 ng/mL or less [87]. Increases in leptin levels before the development of overt GDM have also been reported by others [88]. Moreover, increased leptin levels also occur in the amniotic fluid of pregnant women who subsequently progress to GDM. A 1 ng/mL increase in amniotic leptin levels raises the risk of GDM development by 4%. Amniotic fluid leptin levels and amniotic insulin concentration are directly correlated [89]. In spite of all this evidence, unchanged [90] and decreased levels [91] of leptin were reported in patients with GDM. Differences in disease severity or ethical variations may partially explain these discrepancies.

GDM is considered an aggravation of the inflammatory state that occurs in normal pregnancy and is associated with increased placenta expression of TNF-*α* and IL-6 [92]. These inflammatory cytokines increase the expression of placental leptin mRNA [93]. On the other hand, leptin increases production of TNF-*α* and IL-6 by monocytes. Thus, a vicious cycle develops which perpetuates the inflammatory state and intensifies insulin resistance.

*Resistin*

Resistin is a cysteine-rich peptide hormone that has been detected mostly in tissues involved in the inflammatory processes [94]. Cellular origins of resistin include adipocytes, monocytes, and macrophages [95]. The physiologic role of resistin in obesity and type 2 diabetes mellitus has been the subject of much controversy. Several studies have shown increased expression of resistin in abdominal adipose tissue of obese individuals [96–98] which correlates with the severity of obesity [99] and insulin resistance [100], while others failed to confirm any impact of obesity and insulin resistance on the concentrations of resistin [101, 102]. The detection of a high resistin expression in immune cells [103, 104] implies that it could possibly play a role in the establishment of insulin resistance through effects on inflammation. 

Serum resistin levels in the first and second trimesters of normal pregnancy are similar to those found in nonpregnant women, but levels significantly increase in the third trimester [105]. Additionally, resistin gene expression in term placental tissue is significantly greater than that in chorionic villous tissue in the first trimester [106]. The increased third trimester resistin levels, along with other placental-derived hormones, might contribute to the insulin resistance and postprandial hyperglycemia in the second half of pregnancy. Physiologic concentrations of resistin (10 ng/mL) promote trophoblast glucose uptake, while higher concentrations (50–100 ng/mL) significantly impair it [107]. 

The precise physiologic role of resistin in human pregnancy remains to be determined. Studies of resistin levels during pregnancy complicated with GDM have produced inconsistent results; elevated [43, 108, 109], lower [46, 110], or even unaltered values [45] have all been reported. Lappas et al. showed a biphasic effect of insulin on the release of resistin [111]. Low concentrations of insulin greatly enhance the release of resistin, while it returns to basal levels when the placenta is exposed to higher insulin concentrations, possibly by a downregulation of resistin expression in the presence of high insulin concentration. This biphasic effect of insulin may explain the low resistin levels reported in GDM [110].

*TNF-*α**

Normal pregnancy is accompanied by a proinflammatory environment. TNF-*α*, which is correlated with insulin resistance in obesity, could also play similar roles in GDM and preeclampsia as well. The placenta is the main site of TNF-*α* (and interleukin-6, another inflammatory mediator) production during pregnancy and levels of TNF-*α* peak in late gestation. The vast majority of the TNF-*α* synthesized by the placenta is delivered to maternal circulation with only a small amount to the fetal compartment [26]. The rise in TNF-*α* levels may be related to pregnancy-associated increases insulin resistance [26, 112]. There is strong evidence linking TNF-*α* to downregulation of insulin receptor signaling in cultured adipocytes [113], hepatocytes [114], and skeletal muscles [115]. Importantly, increased TNF-*α* is associated with insulin resistance in obesity [116], aging [117], sepsis [118], and after muscle damage [119]. Studies made *in vitro* report that placental tissues from women with GDM release greater amounts of TNF-*α* in response to a glucose stimulus than those from women with normal glucose tolerance [120]. In this regard, TNF-*α* has been hypothesized to exert an inhibitory effect on insulin secretion and insulin-regulated glucose uptake in GDM, thus contributing to the sustained hyperglycemia [121]. Furthermore, TNF-*α* has been shown to be a significant independent predictor of insulin resistance in GDM [26].

*Visfatin*

Visfatin is another adipokine which is mainly expressed in visceral adipose tissue. It shows insulin-like effects on cultured cells and decreases plasma glucose levels in mice [122]. Its pathophysiological role, along with other adipokines, is largely unknown. Plasma level rises in visfatin increase during obesity, type 2 diabetes, and the metabolic syndrome [122–124] and fluctuate in normal weight pregnant women with peak levels between 19 and 26 weeks and a nadir between 27 and 34 weeks [109]. Some investigators have not observed a relationship between visfatin and visceral fat mass, BMI, or insulin sensitivity [123, 125]. Visfatin expression occurs in human fetal membranes and placenta [126], which is related to mRNA expression of TNF-*α* and IL-6 [127]. Visfatin is also secreted from the human amniotic epithelium and shows antiapoptotic effects on both amniotic epithelial cells and fibroblasts, where it protects them from apoptosis induced by chronic distension, labor, or infection [128]. Increased expression levels of visfatin mRNA in adipose tissue of both pregnant human [126] and animal [129] suggest its participation in energy homeostasis during pregnancy to meet the nutritional demands of fetal growth [130].

There are no consistent results on the plasma levels of visfatin in GDM, as both increased [131–133] and decreased [127, 134–136] concentrations have been reported. Mastorakos et al. reported that visfatin concentrations in the first trimester positively predict insulin sensitivity during the second trimester in nonobese, nondiabetic white women [137]. Furthermore, the immune-modulatory properties of visfatin can significantly affect insulin resistance. Treatment of human fetal membranes with recombinant human visfatin significantly increases levels of some inflammatory cytokines such as IL-1*β*, TNF-*α*, and IL-6, all of which influence insulin sensitivity [138].

*Apelin*

 Apelin is another adipokine secreted from adipocytes [139] and several other tissues [140]. Even though its role in normal physiology has not been described precisely, several functions have been named for this bioactive peptide. Apelin participates in both normal and pathologic angiogeneses [141] which may help in the growth of adipose tissue [142]. Insulin increases apelin synthesis in adipocytes and plasma apelin level rises in obesity associated with insulin resistance [143]. Apelin also reduces blood pressure by enhancing endothelium dependent vasodilation [144]. 

Apelin expression has been demonstrated in human placental tissue [145] and is thought to be required for endothelial cell proliferation and growth of blood vessels [146]. A recent human study reported increased apelin levels in maternal serum of women with GDM [147]. However, further studies are needed to clarify the role of this novel adipokine in normal and complicated pregnancy.

*Chemerin*

Chemerin is another protein that is highly expressed in human adipose tissue, liver, and lung and has a role in adaptive and innate immunity [148]. Chemerin boosts inflammation by stimulating chemotaxis [149]. IL-*β* increases chemerin mRNA expression and secretion from 3T3-L1 derived adipocytes [150]. Since chemerin plays a role in adipocyte differentiation and glucose metabolism, it is also considered an adipokine [151]. Adenoviral small hairpin RNA targeted knockdown of chemerin (or its receptors) impairs differentiation of 3T3-L1 preadipocytes and decreases the expression of lipid and glucose metabolizing genes in adipose tissue [151]. Chemerin level in humans correlates with BMI, plasma lipids, and blood pressure [151]. Increased serum concentration of chemerin occurs in individuals with type 2 diabetes [152]. However, studies aimed at evaluating the role of chemerin in GDM did not demonstrate a clear association between metabolic dysregulation and chemerin levels during GDM [153, 154].

## 3. Role of Exercise in GDM Management

Even though some studies were inconclusive on the benefits of exercise in preventing GDM [155, 156], there is overwhelming evidence suggesting that women who exercise have a considerably lower chance of developing GDM [81, 82, 93, 157]. The Canadian Diabetes Association (CDA) recommends that “Physical activity should be encouraged, with the frequency, type, duration, and intensity tailored to individual obstetric risk” [1]. The American Diabetes Association also suggests “Women without medical or obstetrical contraindications are encouraged to start or continue a program of moderate exercise as part of treatment for GDM” [2]. Participating in any physical activity during the first 20 weeks of pregnancy leads to an approximately 50% risk reduction for GDM [158]. In a prospective cohort study among 21,765 women in the Nurses' Health Study II, Zhang et al. showed that physical activity before pregnancy is associated with a risk reduction in GDM. It is interesting to note that subjects not performing intense exercise but instead engage in brisk walking also enjoy a similar risk reduction [159]. Women who engage in intense physical activity before pregnancy have a 44% and 24% risk reduction for GDM and abnormal glucose tolerance, respectively [160]. In a case controlled study of physical activity in 155 pregnant women with GDM compared with 386 healthy pregnant controls, physical activity before and during pregnancy was associated with a reduced incidence of GDM [158]. 

In spite of these studies, there remain many long-standing myths on the harms of exercise during pregnancy. For instance, some believe that women who are unused to exercise before pregnancy should not start when pregnant, while others suggest that pregnancy means eating for two. In a study of pregnant women in Norway, 55% were recognized as nonexercisers (≤20 minutes of vigorous recreational physical activity at least once a week) in the third trimester and 66.5% reported walking ≤30 minutes per day [161]. Unfortunately, many women reduce their physical activity during pregnancy, resulting in gaining more weight than is recommended. Age, education, working status, health condition, and psychosocial factors such as social modeling and knowledge all determine likelihood of weight gain and a sedentary lifestyle during pregnancy [162].

Aerobic exercise is the recommended type of exercise to prevent excessive weight gain and maintain cardiovascular fitness. A recent study suggests that the amount of exercise for pregnant women should be equivalent to energy expenditure of 16 (ideally 28) metabolic equivalent tasks (METs) per week. This can be achieved by walking 5.1 kilometers every day or using a stationary bicycle for 45 min each day [163]. It is advised that activities such as contact sports be avoided and attention paid to adequate hydration and avoidance of exercising in uncomfortably hot and humid environments [164]. When starting an aerobic exercise program, careful consideration should be given to the intensity of exercise. Most experts suggest exercising to 60%–70% of maximal heart rate for those who were sedentary before pregnancy and 60%–90% of maximal heart rate for those who are well trained. Borg's rating of perceived exertion is another method to assure an ideal intensity of exercise when it is performed on a self-paced base. Scales from 6 to 11 are considered mild, 12 to 14 moderate (or somewhat hard), and 15 to 20 are hard exercises. “Talk test” or physical activity at relaxed strength that allows one to keep up conversation is another method to confirm that intensity of exercise is appropriate and women are not overexerting [82] (Table 1).

### 3.1. Suggested Exercise-Induced Benefits in GDM

There are a few studies of the mechanisms of exercise-induced benefits in GDM. However, because of the similarity between GDM and type 2 diabetes, most of the suggested mechanisms in diabetes can be extrapolated to GDM.

#### 3.1.1. Increased Insulin Sensitivity

At least two distinct pathways are involved in glucose transport; one is stimulated by insulin or insulin mimetics and the other activated by contraction or hypoxia [165–167]. Phosphatidylinositol 3 kinase (PI3-kinase) is involved in the insulin activated (but not contraction activated) pathway [168], while 5'AMP-activated protein kinase (AMPK) participates in contraction activated reactions [169]. Insulin stimulated tyrosine phosphorylation of insulin receptor substrate (IRS), activity of PI3 kinase, and insulin stimulated A*κ*t kinase activity are all diminished in skeletal muscle of obese, diabetic, and GDM patients [92, 170]. Therefore, exercise can provide an alternative way to bypass the impaired insulin signal transduction in muscles of diabetic patients [171]. Regular physical activity improves insulin function and glucose tolerance in healthy individuals [172], patients with obesity [173], insulin resistance [174], and diabetics [175, 176]. Molecular mechanisms for improved glucose clearance and insulin sensitivity following exercise are related to the increased expression and activity of signaling proteins and enzymes that are involved in skeletal glucose and fat metabolism [177, 178]. The biogenesis of glucose transporter isoform 4 (GLUT4), a key enzyme in insulin stimulated glucose uptake by muscle, is increased by exercise training [179, 180]. Biopsies of the vastus lateralis muscle in pregnant women show increased GLUT4 expression in mildly exercise-trained women [181]. The transcriptional factor peroxisome proliferator-activated receptor *γ* coactivator-1 (PGC-1) stimulates GLUT4 expression in addition to stimulating mitochondrial biogenesis and promoting muscle remodeling to a fiber type composition that has greater oxidative capacity and less glycolytic metabolism in nature [182, 183]. 

However, exercise-induced improvement in insulin signaling is not exclusively restricted to increased GLUT4 protein expression, as its concentration is similar in sedentary diabetics and insulin-sensitive control subjects [184, 185]. While exercise increases GLUT4 protein and mRNA in diabetic patients [186], increased postreceptor insulin signaling, especially at the distal step of the insulin PI3-kinase cascade (which results in GLUT4 translocation and glucose uptake), is the main mechanism [178, 187, 188]. Atypical protein kinase C (aPKC) and Akt substrate of 160 kDa (AS160) are among newly characterized insulin signaling molecules [189, 190]. AS160 in the basal nonphosphorylated state acts as an inhibitor for GLUT4 translocation. Insulin stimulates AS160 phosphorylation by Akt on five of six phosphor-Akt substrate motifs, leading to increased GLUT4 membrane trafficking events [191]. The exact mechanisms of aPKC in controlling GLUT4 translocation are still not clear, but some reports suggest that parallel to Akt, activation of aPKC is essential in both the process of translocation and docking/fusion of GLUT4 to the plasma membrane [192].

There are many changes in exogenous insulin requirement and glycemic control after a 4–8-week period of exercise in the last trimester of pregnancy [193–195]. For example, there are reduced levels of glycosylated hemoglobin, fasting, and 1-hour plasma glucose following a six-week arm ergometry in pregnant women with GDM [193]. This exercise protocol was significantly milder, in terms of duration and frequency, than those which have been suggested for diabetic or gravid subjects [196, 197]. In another study of GDM patients unresponsive to dietary therapy, 8 weeks of supervised exercise (50% of VO2max/3 times a week) maintained euglycemia without the need for insulin therapy [195]. It is important that exercise is performed on a chronic basis so as to have a sustained impact on glycemic control, since several studies report a decline in postprandial plasma glucose upon cessation of exercise [198, 199].

#### 3.1.2. Adipokine Changes

Weight reduction in obese subjects, via exercise, results in a lower loss of muscle (compared to fat) than weight loss through diet [200]. Maintaining lean body mass is essential for better glucose transport and fat metabolism. A reduction in fat mass is helpful in increasing adiponectin levels and improving cytokine profiles. Controlling the release and activity of at least two cytokines, TNF-*α* and IL-6, could contribute to the natural protective effects of physical activity. Interleukin-6 (IL-6) is the first cytokine to be released into the circulation during exercise, and its levels increase in an exponential fashion in response to exercise [201]. IL-6 mRNA is upregulated in contracting skeletal muscle [202] and the transcriptional rate of the IL-6 gene is also markedly enhanced by exercise [203]. IL-6 acts as both a proinflammatory and anti-inflammatory cytokine: when secreted by T cells and macrophages, IL-6 stimulates the immune response and boosts inflammatory reactions, while muscle-produced IL-6 exerts anti-inflammatory effects through its inhibitory effects on TNF-*α* and IL-1*β* and activation of interleukin-1 receptor antagonist (IL-1ra) and IL-10 [204]. Exercise-induced increases in plasma IL-6 correlate with the muscle mass involved in exercise activity and also with the mode, duration, and especially the intensity of exercise [205]. Exercise also confers protection against TNF-induced insulin resistance [206]. IL-6 enhances lipid turnover and stimulates lipolysis as well as fat oxidation via activation of AMP-activated protein kinase [207]. The lipolytic effect of IL-6 on fat metabolism was confirmed in two clinical studies of healthy and diabetic subjects [207, 208]. During exercise, IL-6 also increases hepatic glucose production. Glucose ingestion during exercise reduces IL-6 production by muscles, suggesting that IL-6 is released due to the reduction in glycogen levels during endurance exercise and the consequences of adrenergic stimulation of IL-6 gene transcription via protein kinase A activation [209].

The study of Clapp III and Kiess is one the few experiments that evaluated the effects of exercise on metabolic markers during pregnancy [152]. They measured the concentrations of TNF-*α* and leptin in a control group of physically active women and compared this with groups of active and nonactive pregnant subjects. In this experiment, regular weight bearing exercise suppressed the pregnancy-associated changes normally seen in both TNF-*α* and leptin. The authors inferred that leptin reduction is a reflection of decreased fat accretion, and changes in TNF-*α* could be evidence of altered insulin resistance [152]. Even though exercise-induced TNF-*α* changes have been reported by other investigators in both pregnant and nonpregnant subjects [210, 211], there is no consistency in the case of exercise-induced leptin changes. For example, Hopkins et al. [212] reported an increase in maternal leptin from mid to late pregnancy following aerobic exercise. This discrepancy in leptin levels has been observed in nonpregnant individuals as well [213–215].

#### 3.1.3. Oxidative Stress and Antioxidant Effect of Exercise on GDM

One characteristic of pregnancy is the early accumulation of fat depots, followed by increased adipose tissue lipolysis and increased levels of plasma free fatty acids (FFAs) which all enhance insulin resistance [216]. Intramyocellular accumulation of diacylglycerol and subsequent activation of protein kinase C are thought to mediate FFA-stimulated insulin resistance in skeletal muscles. Insulin resistance leads to reduction of tyrosine phosphorylation of the IRS-1 and inhibits activation of PI3 kinase [217]. Increased intramyocellular lipids increase cellular oxidative stress with subsequent generation of ROS, stimulating lipid membrane peroxidative injury of mitochondrial membranes. Oxidative stress inhibits expression of adipokines [218]. Increase in TNF-*α* and IL-6 during diabetes may be due to hyperglycemia related to oxidative stress and inflammation [83]. One of the cornerstone effects of exercise training is to augment the oxidative capacity of skeletal muscles, so that there is an improvement in the rate of whole body fat oxidation [219]. This increase in fat oxidation capacity is partly due to increases in fatty acid transport proteins, leading to increased removal of plasma FFAs [220]. Plasma membrane-associated fatty acid binding proteins (FABPpm) and fatty acid translocase/CD36 (FAT/CD36) are among several key proteins that have been identified as fatty acid transporter proteins in human and animal muscles [221]. Exercise also activates AMPK, which stimulates fatty acid oxidation, glucose uptake, and mitochondrial biogenesis.

There are many studies which have evaluated the role and importance of oxidative stress in pathogenesis of type 2 diabetes; however, this role of oxidative stress in GDM has received much less attention. The term oxidative stress indicates a shift towards a prooxidant environment in the balance between oxidant species formation and antioxidant defenses. Chemical compounds capable of producing potential toxic reactive oxygen species (ROS) are known as prooxidants and antioxidants are compounds detoxifying ROS. Free radicals are reactive chemical species having a single unpaired electron in an outer orbit. This unstable configuration provides energy which is released through reactions with adjacent molecules such as proteins, lipids, carbohydrates, and nucleic acids. The majority of free radicals that damage biological systems are oxygen-free radicals [222].

An antioxidant stabilizes or deactivates free radicals before they attach to cells. Humans have evolved highly complex antioxidant systems (enzymatic and nonenzymatic) that work synergistically, and in combination with each other, to protect cells and organ systems against free radical induced damage. Antioxidants can be endogenously produced substances or can be obtained from exogenous sources, for example, as a part of a diet or as dietary supplements. Endogenous antioxidants play a crucial role in maintaining optimal cellular functions and thus systemic health and well-being. However under conditions which promote oxidative stress, endogenous antioxidants may not be sufficient and dietary antioxidants may be required to maintain optimal cellular functions. The most efficient enzymatic antioxidants involve glutathione peroxidase, catalase and superoxide dismutase. Nonenzymatic antioxidants include vitamins E and C, thiol antioxidants (glutathione, thioredoxin, and lipoic acid), melatonin, carotenoids, natural flavonoids, and other compounds [223].

There are limited data suggesting that oxidative stress may be involved in progression or pathophysiology of GDM. Coughlan et al. reported that the release of 8-isoprostane, along with superoxide dismutase activity and protein carbonyl from human placental explants, is significantly increased in GDM compared to normal placental tissues [224]. They also reported that placentae from women with GDM display a reduced capacity to respond to oxidative stress [225]. Markers of ROS, such as 8-isoprostane, are increased in placenta, subcutaneous adipose tissue, and skeletal muscle in women with GDM [226]. These data are consistent with the hypothesis that oxidative stress may be involved in the progression and/or pathogenesis of GDM. Other related studies suggest that oxidative stress in GDM is also related to an altered antioxidant capacity as well. In a comparison between healthy pregnant women with two groups with diabetes (GDM and type 1 diabetes), Peuchant et al. reported that plasma and erythrocyte free malondialdehyde (MDA) levels were significantly higher, while levels of plasma vitamin E, erythrocyte vitamin A, and glutathione peroxidase (GPX) were lower, in both diabetic (including GDM) subjects [227]. Evidence of lipid peroxidation and protein oxidative damage is also present in the erythrocytes of both mothers with GDM and their newborn infants [228]. In a longitudinal study, Toescu et al. showed evidence for higher serum lipid and lipid hydroperoxide levels and lower corrected antioxidant capacity throughout pregnancy in diabetic women (type 1, 2 and GDM) [229]. 

Exercise training leads to an upregulation of antioxidant defense mechanisms in various tissues, presumably due to increased levels of oxidative stress that occurs during exercise. Exercise-induced production of ROS provokes specific adaptations such as increased antioxidant/oxidative damage repairing enzyme activity, increased resistance to oxidative stress, and lower levels of oxidative damage. Physiological levels of shear stress increase the expression of Cu/Zn SOD in human aortic endothelial cells [230], while endurance training mainly induces Mn-SOD expression [231]. In our experiments with type 2 diabetic mice *(db/db) *we observed a specific downregulation of aortic Mn-SOD following diabetes. Low-intensity exercise increased Cu/Zn-SOD protein production, whereas moderate intensity exercise increased Mn-SOD [232]. Others have also reported such preferential effects of exercise on antioxidant enzyme regulation. For instance, Sankaralinqam et al. reported that arteries from pregnant women involved in low intensity exercise (stretching) had significantly greater expression of the vascular antioxidant enzyme SOD when compared with those who performed moderate intensity exercise (walking) [233]. The effect of exercise on raising the levels of glutathione peroxidase and catalase has also been reported in pregnant women [234].

## 4. Summary

Obesity is reaching epidemic proportions in modern society. Overweight women are at increased risk of several complications during pregnancy, including GDM. Complications of obesity further add to the metabolic changes that promote adipose tissue accretion in early gestation and later onset of insulin resistance. Recent investigations have focused on the role of adipokines or adipocytokines as mediators of insulin resistance. This paper focuses on their role in insulin resistance during pregnancy. Existing data supports the notion that exercise increases insulin sensitivity, possibly by changing the adipokines profile and by upregulating antioxidant defense mechanisms. It is likely that based on current knowledge, regular participation in physical activity could reduce risk profiles for GDM in pregnant women.

## Figures and Tables

**Table 1:** Important facts about recommending exercise to pregnant women [82, 163, 164].

Key points
(i) Exercise is part of healthy lifestyle which should be continued during pregnancy.
(ii) The goal of aerobic exercise in pregnancy is to maintain or improve overall fitness (not training for athletic competitions).
(iii) Contact sports or activities with risks of falling or trauma (snow and water skiing, horseback riding, etc.) should be avoided.
(iv) Exercise does not increase adverse outcomes during pregnancy.
(v) Pregnant women previously unaccustomed to exercise should start gradually and not overexert themselves.
(vi) Women should have self-monitoring exertion. “Easy talk” can be helpful for detection of overexertion.
(vii) Exercise in uncomfortably hot and humid weather should be avoided.
(viii) Achieving 16 MET h/w is a reasonable goal of energy expenditure for those who were previously sedentary.
